# Abnormal osteogenesis in osteoarthritis: gone with the Wnt?

**DOI:** 10.1186/ar3574

**Published:** 2012-02-09

**Authors:** Daniel Lajeunesse, Thomas F Chan, Aline Delalandre, Jean-Pierre Pelletier, Johanne Martel-Pelletier, Élie Abed

**Affiliations:** 1 Unité de recherche en Arthrose, CRCHUM, Université de Montréal, Québec, Canada, H2L 4M1

## Background

Clinical and in vitro studies suggest that subchondral bone sclerosis due to abnormal osteoblast (Ob) functions, is involved in the progression and/or onset of osteoarthritis (OA). Human OA subchondral Ob show a differentiated phenotype, however they fail to mineralize normally. The canonical Wnt/b-catenin signaling pathway (cWnt) plays a key role in osteogenesis by promoting the differentiation and mineralization of Ob. Dickkopfs (DKKs) are potent antagonists whereas R-spondins (Rspo) are newly described agonists that play key roles in cWnt signalling. However, the regulation of DKKs and Rspos in OA Ob remains unknown.

## Materials and methods

We prepared primary human subchondral Ob using the sclerotic medial portion of the tibial plateaus of OA patients undergoing knee arthroplasty, or from tibial plateaus of normal individuals at autopsy. DKK1, DKK2, SOST and Rspo-1 and -2 expression and production were evaluated by qRT-PCR and WB analysis. The regulation of their expression was determined in response to transforming growth factor-ß1 (TGF-ß1) and as a function of the growth of OA Ob. Selective inhibition was performed using siRNA techniques. cWnt signaling was evaluated by measuring target gene expression using the TOPflash Tcf/lef luciferase reporter assay and intracellular ß-catenin levels by WB. Mineralization was evaluated by Alizarin red staining. TGF-ß1 levels were determined by ELISA.

## Results

DKK2 expression and production were elevated in OA Ob compared to normal whereas DKK1 was similar. Rspo2 expression was reduced in OA Ob whereas Rspo1 was similar. TGF-ß1mRNA expression and protein levels were high in OA Ob. TGF-b1 stimulated DKK2 expression and production in Ob whereas it inhibited Rspo2 expression. cWnt signaling was reduced in OA compared to normal Ob. This inhibition was due in part to elevated DKK2 levels and to reduced Rspo-2 levels since correcting DKK2 by siRNA or the addition of Rspo-2 increased cWnt signaling using the TOPflash reporter assay. These treatments also increased ß-catenin levels in OA Ob. Mineralization of OA Ob was reduced compared to normal Ob and was also corrected in part by inhibiting DKK2 or by Rspo2 addition. Both elevated DKK2 and reduced Rspo2 levels contributed to abnormal expression of bone markers by OA Ob.

## Conclusions

These studies demonstrate that elevated antagonist or reduced agonist levels of cWnt signalling interfere in normal Ob function and lead to abnormal mineralization. Since these are secreted soluble proteins, this could lead to potential new avenues of treatment of OA to correct their abnormal bone phenotype and mineralization.

